# 1-Ferrocenyl-3-(3-fluoro­anilino)propan-1-one

**DOI:** 10.1107/S1600536812003510

**Published:** 2012-01-31

**Authors:** Zorica Leka, Sladjana B. Novaković, Anka Pejović, Goran A. Bogdanović, Rastko D. Vukićević

**Affiliations:** a Faculty of Metallurgy and Technology, University of Montenegro, Cetinjski put bb, 81000 Podgorica, Montenegro; b ’Vinča’ Institute of Nuclear Sciences, Laboratory of Theoretical Physics and Condensed Matter Physics, PO Box 522, 11001 Belgrade, Serbia; c Department of Chemistry, Faculty of Science, University of Kragujevac, R. Domanovića 12, 34000 Kragujevac, Serbia

## Abstract

The title ferrocene derivative, [Fe(C_5_H_5_)(C_14_H_13_FNO)], crystallizes in the same space group with similar unit-cell parameters as the derivatives 3-anilino-1-ferrocenylpropan-1-one [Leka *et al.* (2012 ▶). *Acta Cryst.* E**68**, m229] and 1-ferrocenyl-3-(4-methyl­anilino)propan-1-one [Leka *et al.* (2012 ▶). *Acta Cryst.* E**68**, m230]. The dihedral angle between the best planes of the benzene ring and the substituted cyclo­penta­dienyl ring is 83.4 (1)°. The presence of the electronegative fluoro substituent in the *meta* position of the aniline group does not alter the crystal packing compared to the other two derivatives. The molecules are connected into centrosymmetric dimers *via* N—H⋯O hydrogen bonds. In addition, C—H⋯O and C—H⋯N contacts stabilize the crystal packing.

## Related literature

For the physico-chemical properties of ferrocene-based compounds see: Togni & Hayashi (1995 ▶). For related crystal structures and details of the synthesis see: Damljanović *et al.* (2011 ▶); Stevanović *et al.* (2012 ▶); Leka *et al.* (2012*a* ▶,*b* ▶).
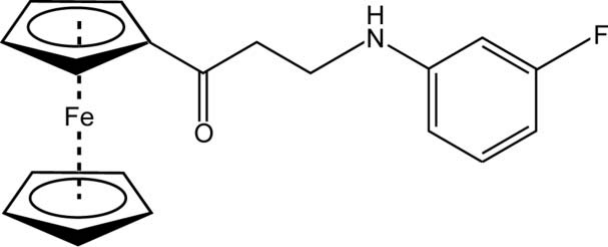


## Experimental

### 

#### Crystal data

[Fe(C_5_H_5_)(C_14_H_13_FNO)]
*M* *_r_* = 351.19
Triclinic, 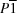

*a* = 7.6602 (4) Å
*b* = 9.6438 (4) Å
*c* = 12.0626 (6) Å
α = 86.548 (4)°
β = 73.590 (4)°
γ = 69.138 (4)°
*V* = 797.95 (7) Å^3^
*Z* = 2
Mo *K*α radiation
μ = 0.96 mm^−1^
*T* = 293 K
0.30 × 0.24 × 0.22 mm

#### Data collection

Oxford Diffraction Xcalibur Sapphire3 Gemini diffractometer
Absorption correction: multi-scan (*CrysAlis PRO*; Oxford Diffraction, 2009 ▶) *T*_min_ = 0.892, *T*_max_ = 1.000
6470 measured reflections
3637 independent reflections
2733 reflections with *I* > 2σ(*I*)
*R*_int_ = 0.039

#### Refinement

*R*[*F* ^2^ > 2σ(*F* ^2^)] = 0.049
*wR*(*F* ^2^) = 0.102
*S* = 1.06
3637 reflections
212 parameters
H atoms treated by a mixture of independent and constrained refinement
Δρ_max_ = 0.32 e Å^−3^
Δρ_min_ = −0.42 e Å^−3^

### 

Data collection: *CrysAlis PRO* (Oxford Diffraction, 2009 ▶); cell refinement: *CrysAlis PRO*; data reduction: *CrysAlis PRO*; program(s) used to solve structure: *SHELXS97* (Sheldrick, 2008 ▶); program(s) used to refine structure: *SHELXL97* (Sheldrick, 2008 ▶); molecular graphics: *ORTEP-3* (Farrugia, 1997 ▶) and *POV-RAY* (Persistence of Vision, 2004 ▶); software used to prepare material for publication: *WinGX* (Farrugia, 1999 ▶), *PLATON* (Spek, 2009 ▶) and *PARST* (Nardelli, 1995 ▶).

## Supplementary Material

Crystal structure: contains datablock(s) I, global. DOI: 10.1107/S1600536812003510/bt5792sup1.cif

Structure factors: contains datablock(s) I. DOI: 10.1107/S1600536812003510/bt5792Isup2.hkl

Additional supplementary materials:  crystallographic information; 3D view; checkCIF report

## Figures and Tables

**Table 1:** Hydrogen-bond geometry (Å, °)

*D*—H⋯*A*	*D*—H	H⋯*A*	*D*⋯*A*	*D*—H⋯*A*
N1—H1*N*⋯O1^i^	0.83 (3)	2.24 (3)	3.049 (3)	165 (3)
C19—H19⋯O1^i^	0.93	2.57	3.342 (3)	141
C4—H4⋯N1^ii^	0.93	2.66	3.517 (3)	153

Symmetry codes: (i) 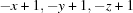; (ii) 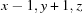.

**Fig. 1.**
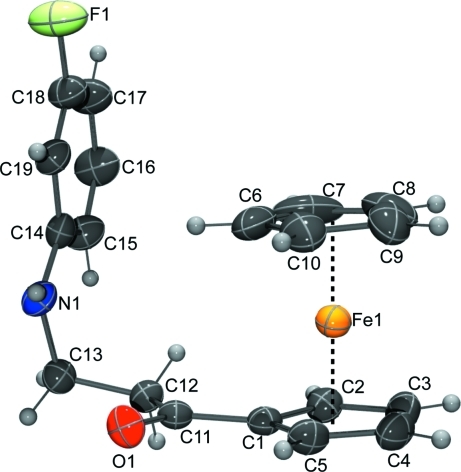
The molecular structure of the title compound, with atom labels and 40% probability displacement ellipsoids for non-H atoms.

**Fig. 2.**
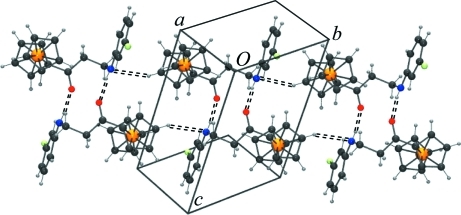
The segment of crystal packing showing the interconnection of the dimers into a chain.

## supplementary crystallographic information

### Comment

As a continuation of our research related to ferrocene containing Mannich bases, we analyzed the crystal structure of 1-Ferrocenyl-3-(3-fluorophenylamino)propan-1-one (I). The present compound (Figure 1) exhibits the pronounced similarity to the previous ones, either in bond lengths and angles as well as in molecular conformation. The mutual orientation of the cyclopentadienyl (Cp) rings within the Fc unit, given by the smallest torsion angle C—C*g*1—C*g*2—C of 4.5° is close to eclipsed one, with small mutual twisting as in the case of 1-Ferrocenyl-3-(phenylamino)propan-1-one (Leka *et al.*, 2012*a*) and 1- Ferrocenyl-3-(*p*-tolylamino)propan-1-one (Leka *et al.*, 2012*b*).

The Cp rings are almost parallel forming the dihedral angle between the Cp ring planes of 1.3 (2)°, while the distances of Fe1 to C*g*1 and C*g*2 centroids are 1.64 and 1.65 Å, respectively. In accordance with previously observed trend the Fe1···C*g* distances toward the substituted Cp ring are 0.01 Å shorter than those toward the unsubstituted ring. The torsion angles within the most flexible, aliphatic part of the molecule (C1—C11—C12—C13—N1) indicate a molecular conformation similar to the previously reported derivatives. The dihedral angle between the best planes of Cp1 and phenyl ring is 83.4 (1)°. It is worth noticing that the presence of the electronegative fuloro substituent on the phenylamino moiety has no influence on the molecule arrangement (Fig. 2), in fact F atom do not participate in any interaction. The closest donor, the cyclopentadienyl C7—H fragment is placed at the distance of 2.69 Å. Molecules exhibit arrangement which is very similar to those observed in phenylamino (Leka *et al.*, 2012*a*) and tolylamino (Leka *et al.*, 2012*b*) derivatives.

### Experimental

The compound was obtained by an aza-Michael addition of the corresponding arylamine to acryloylferrocene. The reaction was performed by microwave (MW) irradiation (500 W/5 min) of a mixture of reactants and montmorillonite K-10, without a solvent as described by Damljanović *et al.* (2011).

### Refinement

H atoms bonded to C atoms were placed at geometrically calculated positions and refined using a riding model. C—H distances were fixed to 0.93 and 0.97 Å from aromatic and methylene C atoms respectively. The *U*_iso_(H) values were equal to 1.2 times *U*_eq_ of the corresponding parent atom. H atom attached to N atom was isotropically refined.

### Crystal data

**Table:**

[Fe(C_5_H_5_)(C_14_H_13_FNO)]	*Z* = 2
*M**_r_* = 351.19	*F*(000) = 364
Triclinic, *P*1	*D*_x_ = 1.462 Mg m^−^^3^
Hall symbol: -P 1	Mo *K*α radiation, λ = 0.71073 Å
*a* = 7.6602 (4) Å	Cell parameters from 2586 reflections
*b* = 9.6438 (4) Å	θ = 3.0–29.0°
*c* = 12.0626 (6) Å	µ = 0.96 mm^−^^1^
α = 86.548 (4)°	*T* = 293 K
β = 73.590 (4)°	Prismatic, orange
γ = 69.138 (4)°	0.30 × 0.24 × 0.22 mm
*V* = 797.95 (7) Å^3^	

### Data collection

**Table:**

Oxford Diffraction Xcalibur Sapphire3 Gemini diffractometer	3637 independent reflections
Radiation source: Enhance (Mo) X-ray Source	2733 reflections with *I* > 2σ(*I*)
graphite	*R*_int_ = 0.039
Detector resolution: 16.3280 pixels mm^-1^	θ_max_ = 29.0°, θ_min_ = 3.0°
ω scans	*h* = −10→10
Absorption correction: multi-scan (CrysAlis PRO; Oxford Diffraction, 2009)	*k* = −12→13
*T*_min_ = 0.892, *T*_max_ = 1.000	*l* = −16→16
6470 measured reflections	

### Refinement

**Table:**

Refinement on *F*^2^	Primary atom site location: structure-invariant direct methods
Least-squares matrix: full	Secondary atom site location: difference Fourier map
*R*[*F*^2^ > 2σ(*F*^2^)] = 0.049	Hydrogen site location: inferred from neighbouring sites
*wR*(*F*^2^) = 0.102	H atoms treated by a mixture of independent and constrained refinement
*S* = 1.06	*w* = 1/[σ^2^(*F*_o_^2^) + (0.0311*P*)^2^ + 0.0409*P*] where *P* = (*F*_o_^2^ + 2*F*_c_^2^)/3
3637 reflections	(Δ/σ)_max_ < 0.001
212 parameters	Δρ_max_ = 0.32 e Å^−^^3^
0 restraints	Δρ_min_ = −0.42 e Å^−^^3^

### Fractional atomic coordinates and isotropic or equivalent isotropic displacement parameters (Å^2^)

**Table:**

	*x*	*y*	*z*	*U*_iso_*/*U*_eq_	
Fe	−0.00273 (5)	0.82243 (4)	0.71263 (3)	0.04303 (14)	
F1	0.7892 (3)	0.3862 (3)	0.90812 (18)	0.0882 (7)	
O1	0.2719 (2)	0.5199 (2)	0.48403 (16)	0.0499 (5)	
N1	0.4911 (3)	0.2934 (3)	0.6422 (2)	0.0449 (6)	
C1	−0.0198 (3)	0.6710 (3)	0.6119 (2)	0.0372 (6)	
C2	−0.1753 (3)	0.7020 (3)	0.7169 (2)	0.0448 (7)	
H2	−0.1983	0.6335	0.7714	0.054*	
C3	−0.2873 (4)	0.8550 (3)	0.7229 (3)	0.0536 (8)	
H3	−0.3970	0.9051	0.7823	0.064*	
C4	−0.2040 (4)	0.9184 (3)	0.6233 (3)	0.0560 (8)	
H4	−0.2504	1.0178	0.6057	0.067*	
C5	−0.0400 (4)	0.8082 (3)	0.5550 (2)	0.0473 (7)	
H5	0.0413	0.8216	0.4850	0.057*	
C6	0.2666 (5)	0.7445 (4)	0.7374 (4)	0.0795 (12)	
H6	0.3657	0.6561	0.7055	0.095*	
C7	0.1231 (8)	0.7621 (6)	0.8431 (4)	0.0967 (15)	
H7	0.1092	0.6879	0.8941	0.116*	
C8	0.0042 (6)	0.9130 (6)	0.8577 (4)	0.0878 (12)	
H8	−0.1030	0.9561	0.9206	0.105*	
C9	0.0721 (5)	0.9861 (4)	0.7643 (4)	0.0719 (10)	
H9	0.0192	1.0871	0.7533	0.086*	
C10	0.2342 (4)	0.8836 (4)	0.6881 (3)	0.0671 (9)	
H10	0.3074	0.9040	0.6176	0.081*	
C11	0.1432 (3)	0.5307 (3)	0.5732 (2)	0.0363 (6)	
C12	0.1427 (3)	0.3981 (3)	0.6467 (2)	0.0404 (6)	
H12A	0.0391	0.3664	0.6403	0.048*	
H12B	0.1158	0.4275	0.7270	0.048*	
C13	0.3347 (3)	0.2684 (3)	0.6115 (2)	0.0443 (7)	
H13A	0.3168	0.1803	0.6483	0.053*	
H13B	0.3718	0.2496	0.5285	0.053*	
C14	0.5010 (3)	0.2932 (3)	0.7547 (2)	0.0397 (6)	
C15	0.3909 (4)	0.2347 (3)	0.8445 (3)	0.0551 (8)	
H15	0.2988	0.2008	0.8311	0.066*	
C16	0.4193 (4)	0.2273 (4)	0.9534 (3)	0.0674 (10)	
H16	0.3464	0.1870	1.0123	0.081*	
C17	0.5519 (4)	0.2778 (4)	0.9769 (3)	0.0672 (9)	
H17	0.5706	0.2723	1.0502	0.081*	
C18	0.6549 (4)	0.3361 (4)	0.8883 (3)	0.0553 (8)	
C19	0.6334 (3)	0.3462 (3)	0.7795 (2)	0.0458 (7)	
H19	0.7066	0.3882	0.7222	0.055*	
H1N	0.541 (4)	0.349 (3)	0.601 (2)	0.044 (8)*	

### Atomic displacement parameters (Å^2^)

**Table:**

	*U*^11^	*U*^22^	*U*^33^	*U*^12^	*U*^13^	*U*^23^
Fe	0.0435 (2)	0.0423 (2)	0.0477 (3)	−0.01725 (18)	−0.01646 (18)	0.00022 (17)
F1	0.0864 (13)	0.1340 (19)	0.0769 (15)	−0.0679 (14)	−0.0380 (11)	0.0171 (13)
O1	0.0517 (11)	0.0520 (11)	0.0418 (12)	−0.0225 (9)	−0.0014 (9)	0.0042 (9)
N1	0.0368 (12)	0.0494 (14)	0.0487 (16)	−0.0169 (11)	−0.0119 (11)	0.0105 (12)
C1	0.0404 (13)	0.0377 (13)	0.0416 (16)	−0.0184 (11)	−0.0182 (12)	0.0036 (11)
C2	0.0397 (13)	0.0473 (16)	0.0511 (18)	−0.0210 (13)	−0.0116 (12)	0.0060 (13)
C3	0.0384 (14)	0.0529 (18)	0.065 (2)	−0.0101 (14)	−0.0133 (14)	−0.0049 (15)
C4	0.0571 (17)	0.0424 (16)	0.071 (2)	−0.0112 (15)	−0.0320 (16)	0.0091 (15)
C5	0.0583 (16)	0.0459 (15)	0.0442 (17)	−0.0215 (14)	−0.0224 (14)	0.0122 (13)
C6	0.066 (2)	0.065 (2)	0.123 (4)	−0.0124 (19)	−0.061 (2)	−0.011 (2)
C7	0.143 (4)	0.124 (4)	0.089 (3)	−0.090 (3)	−0.087 (3)	0.050 (3)
C8	0.086 (3)	0.131 (4)	0.062 (3)	−0.058 (3)	−0.013 (2)	−0.030 (3)
C9	0.0600 (19)	0.061 (2)	0.100 (3)	−0.0257 (17)	−0.0199 (19)	−0.023 (2)
C10	0.0514 (17)	0.078 (2)	0.081 (3)	−0.0330 (18)	−0.0153 (17)	−0.0121 (19)
C11	0.0393 (13)	0.0392 (14)	0.0380 (15)	−0.0202 (11)	−0.0145 (12)	0.0032 (11)
C12	0.0373 (13)	0.0434 (15)	0.0432 (16)	−0.0184 (12)	−0.0109 (11)	0.0063 (12)
C13	0.0461 (14)	0.0382 (14)	0.0502 (18)	−0.0169 (12)	−0.0136 (13)	0.0034 (12)
C14	0.0323 (12)	0.0375 (14)	0.0425 (16)	−0.0076 (11)	−0.0070 (11)	0.0054 (11)
C15	0.0498 (16)	0.066 (2)	0.059 (2)	−0.0323 (15)	−0.0171 (15)	0.0151 (16)
C16	0.0664 (19)	0.092 (3)	0.050 (2)	−0.0414 (19)	−0.0132 (16)	0.0199 (18)
C17	0.070 (2)	0.096 (3)	0.044 (2)	−0.037 (2)	−0.0199 (16)	0.0094 (18)
C18	0.0460 (15)	0.069 (2)	0.057 (2)	−0.0261 (15)	−0.0169 (14)	0.0030 (16)
C19	0.0372 (13)	0.0527 (17)	0.0475 (18)	−0.0185 (13)	−0.0098 (12)	0.0090 (13)

### Geometric parameters (Å, °)

**Table:**

Fe—C1	2.015 (3)	C6—C7	1.402 (5)
Fe—C5	2.021 (3)	C6—H6	0.9300
Fe—C7	2.023 (3)	C7—C8	1.405 (6)
Fe—C8	2.024 (4)	C7—H7	0.9300
Fe—C6	2.031 (3)	C8—C9	1.368 (5)
Fe—C2	2.036 (3)	C8—H8	0.9300
Fe—C10	2.042 (3)	C9—C10	1.398 (4)
Fe—C9	2.042 (3)	C9—H9	0.9300
Fe—C4	2.048 (3)	C10—H10	0.9300
Fe—C3	2.057 (3)	C11—C12	1.511 (3)
F1—C18	1.362 (3)	C12—C13	1.520 (3)
O1—C11	1.217 (3)	C12—H12A	0.9700
N1—C14	1.380 (4)	C12—H12B	0.9700
N1—C13	1.448 (3)	C13—H13A	0.9700
N1—H1N	0.83 (3)	C13—H13B	0.9700
C1—C5	1.432 (3)	C14—C19	1.392 (4)
C1—C2	1.432 (3)	C14—C15	1.397 (4)
C1—C11	1.467 (3)	C15—C16	1.385 (4)
C2—C3	1.412 (4)	C15—H15	0.9300
C2—H2	0.9300	C16—C17	1.371 (4)
C3—C4	1.408 (4)	C16—H16	0.9300
C3—H3	0.9300	C17—C18	1.359 (4)
C4—C5	1.399 (4)	C17—H17	0.9300
C4—H4	0.9300	C18—C19	1.362 (4)
C5—H5	0.9300	C19—H19	0.9300
C6—C10	1.401 (5)		
			
C1—Fe—C5	41.55 (10)	Fe—C4—H4	127.0
C1—Fe—C7	121.77 (16)	C4—C5—C1	107.9 (2)
C5—Fe—C7	157.2 (2)	C4—C5—Fe	70.97 (17)
C1—Fe—C8	157.73 (16)	C1—C5—Fe	69.04 (15)
C5—Fe—C8	159.81 (17)	C4—C5—H5	126.1
C7—Fe—C8	40.61 (17)	C1—C5—H5	126.1
C1—Fe—C6	107.95 (13)	Fe—C5—H5	125.5
C5—Fe—C6	121.21 (15)	C10—C6—C7	108.0 (3)
C7—Fe—C6	40.47 (16)	C10—C6—Fe	70.29 (17)
C8—Fe—C6	67.65 (16)	C7—C6—Fe	69.46 (19)
C1—Fe—C2	41.40 (10)	C10—C6—H6	126.0
C5—Fe—C2	69.20 (11)	C7—C6—H6	126.0
C7—Fe—C2	108.79 (14)	Fe—C6—H6	125.8
C8—Fe—C2	122.24 (15)	C6—C7—C8	107.1 (3)
C6—Fe—C2	126.19 (13)	C6—C7—Fe	70.07 (19)
C1—Fe—C10	124.64 (12)	C8—C7—Fe	69.7 (2)
C5—Fe—C10	106.96 (13)	C6—C7—H7	126.5
C7—Fe—C10	67.81 (16)	C8—C7—H7	126.5
C8—Fe—C10	67.08 (15)	Fe—C7—H7	125.3
C6—Fe—C10	40.25 (13)	C9—C8—C7	108.8 (4)
C2—Fe—C10	162.67 (12)	C9—C8—Fe	71.0 (2)
C1—Fe—C9	161.18 (13)	C7—C8—Fe	69.6 (2)
C5—Fe—C9	123.84 (14)	C9—C8—H8	125.6
C7—Fe—C9	67.37 (16)	C7—C8—H8	125.6
C8—Fe—C9	39.32 (15)	Fe—C8—H8	125.3
C6—Fe—C9	67.32 (14)	C8—C9—C10	108.6 (4)
C2—Fe—C9	156.06 (13)	C8—C9—Fe	69.6 (2)
C10—Fe—C9	40.02 (13)	C10—C9—Fe	69.97 (19)
C1—Fe—C4	68.51 (11)	C8—C9—H9	125.7
C5—Fe—C4	40.20 (11)	C10—C9—H9	125.7
C7—Fe—C4	161.8 (2)	Fe—C9—H9	126.3
C8—Fe—C4	124.67 (16)	C9—C10—C6	107.5 (3)
C6—Fe—C4	155.86 (16)	C9—C10—Fe	70.01 (18)
C2—Fe—C4	67.94 (11)	C6—C10—Fe	69.46 (19)
C10—Fe—C4	120.74 (14)	C9—C10—H10	126.2
C9—Fe—C4	107.89 (14)	C6—C10—H10	126.2
C1—Fe—C3	68.74 (11)	Fe—C10—H10	125.9
C5—Fe—C3	68.21 (12)	O1—C11—C1	121.5 (2)
C7—Fe—C3	125.88 (17)	O1—C11—C12	121.0 (2)
C8—Fe—C3	108.67 (14)	C1—C11—C12	117.5 (2)
C6—Fe—C3	162.91 (15)	C11—C12—C13	112.7 (2)
C2—Fe—C3	40.37 (10)	C11—C12—H12A	109.1
C10—Fe—C3	155.42 (13)	C13—C12—H12A	109.1
C9—Fe—C3	121.22 (12)	C11—C12—H12B	109.1
C4—Fe—C3	40.10 (11)	C13—C12—H12B	109.1
C14—N1—C13	122.9 (2)	H12A—C12—H12B	107.8
C14—N1—H1N	114 (2)	N1—C13—C12	113.3 (2)
C13—N1—H1N	116.5 (18)	N1—C13—H13A	108.9
C5—C1—C2	107.1 (2)	C12—C13—H13A	108.9
C5—C1—C11	125.0 (2)	N1—C13—H13B	108.9
C2—C1—C11	127.6 (2)	C12—C13—H13B	108.9
C5—C1—Fe	69.42 (16)	H13A—C13—H13B	107.7
C2—C1—Fe	70.07 (15)	N1—C14—C19	119.0 (2)
C11—C1—Fe	121.02 (16)	N1—C14—C15	122.8 (3)
C3—C2—C1	107.8 (2)	C19—C14—C15	118.1 (3)
C3—C2—Fe	70.61 (16)	C16—C15—C14	119.8 (3)
C1—C2—Fe	68.53 (14)	C16—C15—H15	120.1
C3—C2—H2	126.1	C14—C15—H15	120.1
C1—C2—H2	126.1	C17—C16—C15	121.9 (3)
Fe—C2—H2	126.3	C17—C16—H16	119.1
C4—C3—C2	108.0 (2)	C15—C16—H16	119.1
C4—C3—Fe	69.62 (15)	C18—C17—C16	117.0 (3)
C2—C3—Fe	69.02 (14)	C18—C17—H17	121.5
C4—C3—H3	126.0	C16—C17—H17	121.5
C2—C3—H3	126.0	C17—C18—F1	118.7 (3)
Fe—C3—H3	127.0	C17—C18—C19	123.7 (3)
C5—C4—C3	109.1 (2)	F1—C18—C19	117.5 (3)
C5—C4—Fe	68.83 (15)	C18—C19—C14	119.5 (3)
C3—C4—Fe	70.28 (15)	C18—C19—H19	120.3
C5—C4—H4	125.4	C14—C19—H19	120.3
C3—C4—H4	125.4		
			
C7—Fe—C1—C5	−159.4 (2)	C9—Fe—C6—C10	−37.7 (2)
C8—Fe—C1—C5	168.2 (3)	C4—Fe—C6—C10	45.5 (4)
C6—Fe—C1—C5	−117.1 (2)	C3—Fe—C6—C10	−161.3 (4)
C2—Fe—C1—C5	118.1 (2)	C1—Fe—C6—C7	−118.2 (3)
C10—Fe—C1—C5	−75.8 (2)	C5—Fe—C6—C7	−161.9 (2)
C9—Fe—C1—C5	−44.9 (4)	C8—Fe—C6—C7	38.5 (2)
C4—Fe—C1—C5	37.50 (16)	C2—Fe—C6—C7	−76.0 (3)
C3—Fe—C1—C5	80.70 (16)	C10—Fe—C6—C7	118.9 (3)
C5—Fe—C1—C2	−118.1 (2)	C9—Fe—C6—C7	81.2 (3)
C7—Fe—C1—C2	82.6 (2)	C4—Fe—C6—C7	164.4 (3)
C8—Fe—C1—C2	50.1 (4)	C3—Fe—C6—C7	−42.3 (6)
C6—Fe—C1—C2	124.83 (19)	C10—C6—C7—C8	−0.2 (4)
C10—Fe—C1—C2	166.12 (17)	Fe—C6—C7—C8	−60.2 (2)
C9—Fe—C1—C2	−163.0 (3)	C10—C6—C7—Fe	60.0 (2)
C4—Fe—C1—C2	−80.57 (16)	C1—Fe—C7—C6	80.4 (2)
C3—Fe—C1—C2	−37.36 (15)	C5—Fe—C7—C6	43.3 (5)
C5—Fe—C1—C11	119.3 (3)	C8—Fe—C7—C6	−117.8 (3)
C7—Fe—C1—C11	−40.1 (3)	C2—Fe—C7—C6	124.2 (2)
C8—Fe—C1—C11	−72.6 (4)	C10—Fe—C7—C6	−37.6 (2)
C6—Fe—C1—C11	2.1 (3)	C9—Fe—C7—C6	−81.1 (2)
C2—Fe—C1—C11	−122.7 (3)	C4—Fe—C7—C6	−159.4 (4)
C10—Fe—C1—C11	43.4 (3)	C3—Fe—C7—C6	165.9 (2)
C9—Fe—C1—C11	74.3 (4)	C1—Fe—C7—C8	−161.8 (2)
C4—Fe—C1—C11	156.7 (2)	C5—Fe—C7—C8	161.1 (3)
C3—Fe—C1—C11	−160.0 (2)	C6—Fe—C7—C8	117.8 (3)
C5—C1—C2—C3	0.1 (3)	C2—Fe—C7—C8	−118.0 (2)
C11—C1—C2—C3	174.3 (2)	C10—Fe—C7—C8	80.2 (3)
Fe—C1—C2—C3	59.91 (19)	C9—Fe—C7—C8	36.7 (2)
C5—C1—C2—Fe	−59.81 (18)	C4—Fe—C7—C8	−41.6 (6)
C11—C1—C2—Fe	114.4 (3)	C3—Fe—C7—C8	−76.3 (3)
C1—Fe—C2—C3	−119.2 (2)	C6—C7—C8—C9	−0.1 (4)
C5—Fe—C2—C3	−80.42 (18)	Fe—C7—C8—C9	−60.5 (3)
C7—Fe—C2—C3	123.8 (2)	C6—C7—C8—Fe	60.4 (2)
C8—Fe—C2—C3	80.9 (2)	C1—Fe—C8—C9	163.9 (3)
C6—Fe—C2—C3	165.4 (2)	C5—Fe—C8—C9	−39.3 (5)
C10—Fe—C2—C3	−160.7 (4)	C7—Fe—C8—C9	119.4 (3)
C9—Fe—C2—C3	47.4 (4)	C6—Fe—C8—C9	81.0 (2)
C4—Fe—C2—C3	−37.11 (17)	C2—Fe—C8—C9	−159.27 (18)
C5—Fe—C2—C1	38.76 (14)	C10—Fe—C8—C9	37.3 (2)
C7—Fe—C2—C1	−117.1 (2)	C4—Fe—C8—C9	−75.2 (3)
C8—Fe—C2—C1	−159.89 (19)	C3—Fe—C8—C9	−116.8 (2)
C6—Fe—C2—C1	−75.4 (2)	C1—Fe—C8—C7	44.5 (5)
C10—Fe—C2—C1	−41.5 (5)	C5—Fe—C8—C7	−158.7 (4)
C9—Fe—C2—C1	166.5 (3)	C6—Fe—C8—C7	−38.4 (2)
C4—Fe—C2—C1	82.07 (16)	C2—Fe—C8—C7	81.3 (3)
C3—Fe—C2—C1	119.2 (2)	C10—Fe—C8—C7	−82.1 (3)
C1—C2—C3—C4	0.2 (3)	C9—Fe—C8—C7	−119.4 (3)
Fe—C2—C3—C4	58.9 (2)	C4—Fe—C8—C7	165.4 (2)
C1—C2—C3—Fe	−58.60 (18)	C3—Fe—C8—C7	123.8 (3)
C1—Fe—C3—C4	−81.48 (19)	C7—C8—C9—C10	0.4 (4)
C5—Fe—C3—C4	−36.66 (18)	Fe—C8—C9—C10	−59.3 (2)
C7—Fe—C3—C4	164.0 (2)	C7—C8—C9—Fe	59.6 (3)
C8—Fe—C3—C4	122.1 (2)	C1—Fe—C9—C8	−160.9 (3)
C6—Fe—C3—C4	−163.4 (5)	C5—Fe—C9—C8	164.7 (2)
C2—Fe—C3—C4	−119.8 (3)	C7—Fe—C9—C8	−37.9 (2)
C10—Fe—C3—C4	46.5 (4)	C6—Fe—C9—C8	−81.9 (3)
C9—Fe—C3—C4	80.7 (2)	C2—Fe—C9—C8	47.5 (4)
C1—Fe—C3—C2	38.28 (16)	C10—Fe—C9—C8	−119.9 (3)
C5—Fe—C3—C2	83.11 (18)	C4—Fe—C9—C8	123.3 (2)
C7—Fe—C3—C2	−76.3 (3)	C3—Fe—C9—C8	81.4 (3)
C8—Fe—C3—C2	−118.2 (2)	C1—Fe—C9—C10	−41.1 (5)
C6—Fe—C3—C2	−43.6 (5)	C5—Fe—C9—C10	−75.4 (3)
C10—Fe—C3—C2	166.3 (3)	C7—Fe—C9—C10	82.0 (3)
C9—Fe—C3—C2	−159.6 (2)	C8—Fe—C9—C10	119.9 (3)
C4—Fe—C3—C2	119.8 (3)	C6—Fe—C9—C10	37.9 (2)
C2—C3—C4—C5	−0.5 (3)	C2—Fe—C9—C10	167.4 (3)
Fe—C3—C4—C5	58.0 (2)	C4—Fe—C9—C10	−116.8 (2)
C2—C3—C4—Fe	−58.5 (2)	C3—Fe—C9—C10	−158.7 (2)
C1—Fe—C4—C5	−38.72 (16)	C8—C9—C10—C6	−0.5 (4)
C7—Fe—C4—C5	−166.6 (4)	Fe—C9—C10—C6	−59.6 (2)
C8—Fe—C4—C5	161.7 (2)	C8—C9—C10—Fe	59.1 (2)
C6—Fe—C4—C5	47.3 (4)	C7—C6—C10—C9	0.4 (4)
C2—Fe—C4—C5	−83.46 (18)	Fe—C6—C10—C9	59.9 (2)
C10—Fe—C4—C5	79.8 (2)	C7—C6—C10—Fe	−59.5 (2)
C9—Fe—C4—C5	121.65 (19)	C1—Fe—C10—C9	165.1 (2)
C3—Fe—C4—C5	−120.8 (3)	C5—Fe—C10—C9	122.8 (2)
C1—Fe—C4—C3	82.09 (18)	C7—Fe—C10—C9	−80.8 (3)
C5—Fe—C4—C3	120.8 (3)	C8—Fe—C10—C9	−36.6 (2)
C7—Fe—C4—C3	−45.8 (5)	C6—Fe—C10—C9	−118.6 (3)
C8—Fe—C4—C3	−77.5 (2)	C2—Fe—C10—C9	−162.7 (4)
C6—Fe—C4—C3	168.2 (3)	C4—Fe—C10—C9	81.2 (3)
C2—Fe—C4—C3	37.35 (18)	C3—Fe—C10—C9	48.3 (4)
C10—Fe—C4—C3	−159.44 (18)	C1—Fe—C10—C6	−76.3 (3)
C9—Fe—C4—C3	−117.5 (2)	C5—Fe—C10—C6	−118.6 (2)
C3—C4—C5—C1	0.6 (3)	C7—Fe—C10—C6	37.8 (2)
Fe—C4—C5—C1	59.42 (18)	C8—Fe—C10—C6	82.0 (3)
C3—C4—C5—Fe	−58.8 (2)	C2—Fe—C10—C6	−44.1 (5)
C2—C1—C5—C4	−0.4 (3)	C9—Fe—C10—C6	118.6 (3)
C11—C1—C5—C4	−174.8 (2)	C4—Fe—C10—C6	−160.2 (2)
Fe—C1—C5—C4	−60.6 (2)	C3—Fe—C10—C6	166.9 (3)
C2—C1—C5—Fe	60.22 (18)	C5—C1—C11—O1	−3.5 (4)
C11—C1—C5—Fe	−114.1 (2)	C2—C1—C11—O1	−176.7 (3)
C1—Fe—C5—C4	118.7 (2)	Fe—C1—C11—O1	−88.9 (3)
C7—Fe—C5—C4	169.2 (3)	C5—C1—C11—C12	178.2 (2)
C8—Fe—C5—C4	−48.4 (4)	C2—C1—C11—C12	5.0 (4)
C6—Fe—C5—C4	−159.41 (19)	Fe—C1—C11—C12	92.8 (2)
C2—Fe—C5—C4	80.03 (18)	O1—C11—C12—C13	12.5 (4)
C10—Fe—C5—C4	−117.84 (19)	C1—C11—C12—C13	−169.1 (2)
C9—Fe—C5—C4	−77.3 (2)	C14—N1—C13—C12	72.1 (3)
C3—Fe—C5—C4	36.57 (17)	C11—C12—C13—N1	71.8 (3)
C7—Fe—C5—C1	50.6 (4)	C13—N1—C14—C19	−167.6 (2)
C8—Fe—C5—C1	−167.0 (3)	C13—N1—C14—C15	15.9 (4)
C6—Fe—C5—C1	81.9 (2)	N1—C14—C15—C16	174.8 (3)
C2—Fe—C5—C1	−38.62 (14)	C19—C14—C15—C16	−1.7 (4)
C10—Fe—C5—C1	123.50 (17)	C14—C15—C16—C17	0.8 (5)
C9—Fe—C5—C1	164.08 (15)	C15—C16—C17—C18	0.2 (5)
C4—Fe—C5—C1	−118.7 (2)	C16—C17—C18—F1	−179.4 (3)
C3—Fe—C5—C1	−82.09 (16)	C16—C17—C18—C19	−0.2 (5)
C1—Fe—C6—C10	122.8 (2)	C17—C18—C19—C14	−0.7 (5)
C5—Fe—C6—C10	79.2 (2)	F1—C18—C19—C14	178.5 (3)
C7—Fe—C6—C10	−118.9 (3)	N1—C14—C19—C18	−175.0 (2)
C8—Fe—C6—C10	−80.4 (3)	C15—C14—C19—C18	1.6 (4)
C2—Fe—C6—C10	165.10 (19)		

### Hydrogen-bond geometry (Å, °)

**Table:**

*D*—H···*A*	*D*—H	H···*A*	*D*···*A*	*D*—H···*A*
N1—H1N···O1^i^	0.83 (3)	2.24 (3)	3.049 (3)	165 (3)
C19—H19···O1^i^	0.93	2.57	3.342 (3)	141
C4—H4···N1^ii^	0.93	2.66	3.517 (3)	153

Symmetry codes: (i) −*x*+1, −*y*+1, −*z*+1; (ii) *x*−1, *y*+1, *z*.
